# The Mouse Models of Human Cancer database (MMHCdb)

**DOI:** 10.1242/dmm.050001

**Published:** 2023-04-14

**Authors:** Dale A. Begley, Debra M. Krupke, John P. Sundberg, Emily L. Jocoy, Joel E. Richardson, Steven B. Neuhauser, Carol J. Bult

**Affiliations:** The Jackson Laboratory, 600 Main Street, Bar Harbor, ME 04609, USA

**Keywords:** Cancer, Database, Genetic background, Mouse models, Nomenclature

## Abstract

The laboratory mouse has served for decades as an informative animal model system for investigating the genetic and genomic basis of cancer in humans. Although thousands of mouse models have been generated, compiling and aggregating relevant data and knowledge about these models is hampered by a general lack of compliance, in the published literature, with nomenclature and annotation standards for genes, alleles, mouse strains and cancer types. The Mouse Models of Human Cancer database (MMHCdb) is an expertly curated, comprehensive knowledgebase of diverse types of mouse models of human cancer, including inbred mouse strains, genetically engineered mouse models, patient-derived xenografts, and mouse genetic diversity panels such as the Collaborative Cross. The MMHCdb is a FAIR-compliant knowledgebase that enforces nomenclature and annotation standards, and supports the completeness and accuracy of searches for mouse models of human cancer and associated data. The resource facilitates the analysis of the impact of genetic background on the incidence and presentation of different tumor types, and aids in the assessment of different mouse strains as models of human cancer biology and treatment response.

## Teaser

**Summary:** The Mouse Models of Human Cancer database (MMHCdb) is a comprehensive resource for knowledge about diverse *in vivo* mouse models of human cancer.

## INTRODUCTION

The laboratory mouse has long been an important model system for the study of the genetic and genomic basis of human disease and biology. Inbred mice have been used to study the pathobiology of human disease since the early 1900s (Naf et al., 2002). Investigations on the effects of genetic background on tumor incidence and predisposition were among the earliest lines of cancer research using inbred mice (Little and Tyzzer, 1916; Tyzzer, 1909). Although mouse models do not fully recapitulate all aspects of human biology, their genetic and physiological similarities to humans and their experimental tractability have yielded mechanistic insights into human diseases and novel therapeutic strategies (Sharpless and Depinho, 2006; Kopetz et al., 2012; McGonigle and Ruggeri, 2014; Sundberg et al., 2013). The landscape of mouse models of human cancer has evolved dramatically over the years in response to the advent of precision genome-editing technologies, improved immunodeficient hosts for xenograft models, and the availability of panels of genetically diverse mice (Kopetz et al., 2012; Justice et al., 2011; Abate-Shen and Pandolfi, 2013; Liu et al., 2017; Threadgill et al., 2011).

Managing the knowledge about the ever-changing nature of mouse models of human cancer, and the growing corpus of publications and heterogenous data associated with these models, is key to ensuring that an appropriate model is used for a specific research question or application. However, searching for and aggregating information about mouse models can be a daunting challenge, in part because well-established nomenclature guidelines and persistent identifiers for genes, alleles and strains are not often used in the published scientific literature or by data repositories. Using natural language processing of scientific journal articles, Chen et al. (2005) found that up to 85.1% of extracted mouse gene names were ambiguous. They also found that 74.7% of gene symbols in 50 randomly selected abstracts were synonyms instead of the official nomenclature. For example, the name and symbol for the mouse gene transformation related protein 53 (*Trp53*) were never used, whereas the synonym p53 was used frequently. The synonyms for the gene *Erbb2* include *Her2* and *Neu*, which are commonly used in the literature. The official symbol and/or persistent gene identifier for *Erbb2* (e.g. MGI:95410 or NCBI Gene ID:13866) are rarely used in publications. The use of official nomenclature is particularly important for unambiguous identification of models in cases in which gene symbols can refer to multiple different genes or are synonyms for genes in other species. The symbol P60, for example, is a synonym for mouse genes *Ppr1* and *Stip1*, and a synonym for human genes *ARHGEF5*, *SRC*, *SQSTM1*, *IFIT3*, *IFIT3B* and *TNFRSF1A*. P130 is a synonym for the mouse genes *Nolc1*, *Rab3gap1* and *Rb12*. Simple keyword searches by PubMed or Google using ambiguous gene symbols require users to resolve nomenclature ambiguity manually.

Another common practice in the literature that complicates aggregation of knowledge for a specific model and comparison of data across mouse models is the use of generic symbols for knockout and conditional alleles without reference to the official allele name and symbol (e.g. P53^−^ instead of a specific allele such as *Trp53^tm1Tyj^*). The Mouse Models of Human Cancer database (MMHCdb) contains records for 403 mouse strains that were reported in the literature as P53^−^. The lack of official nomenclature and persistent identifiers complicates the ability of researchers to quickly determine whether an engineered allele is germline or induced somatically. Often the strain background of the mouse model is not indicated, which is particularly problematic for reproducibility of research results as the same allele on different genetic backgrounds can result in very different cancer phenotypes (Chan et al., 2021; Hunter et al., 2018; Levine, 2017; Lifsted et al., 1998; Reilly, 2016; Svendsen et al., 2011). MMHCdb curators address this problem by using references in the source publication to identify the precise strain and allele and make corrections in the database record. Unofficial nomenclatures for these entities are recorded as synonyms. If the references are ambiguous, then the curators contact the communicating author to confirm the correct official strain/allele nomenclature. If a strain background is unable to be determined, the strain is given the designation of ‘[not specified]’. If an allele reported in the literature cannot be resolved to an official symbol, the data for the model are not included in the MMHCdb.

The MMHCdb was launched in 1998 as the Mouse Tumor Biology database (MTB), with the goal of providing web-based access to published and unpublished data on the pathobiology of cancer in genetically defined strains of laboratory mice (Bult et al., 1999). The MMHCdb is a contributing data resource to the Mouse Genome Informatics (MGI) consortium hosted at The Jackson Laboratory (Ringwald et al., 2022). The MMHCdb leverages annotation standards pioneered by MGI, including standardized genetic nomenclature for genes and mouse strains and bio-ontologies for annotation of gene function and phenotype. The initial focus of the database was on inbred and hybrid mouse strains and genetically engineered mouse models (GEMMs). As the types of mouse models have changed, so has the range of *in vivo* models represented in the resource (Bult et al., 2015). In 2019 the database name was changed from the ‘Mouse Tumor Biology database’ to the ‘Mouse Models of Human Cancer database’ to better reflect the translational and clinical relevance of mouse models. In 2020, the look and feel of the website was overhauled, and advanced search capabilities using faceted search interfaces were implemented.

The MMHCdb is unique among other open-access community databases and knowledgebases centered on the laboratory mouse because of the breadth of cancer types represented in the resource and the detail provided about the types and frequencies of tumors observed in different cancer models. The Mouse Genome Database (MGD) (Blake et al., 2021) is the source of official nomenclature for genes, alleles and genotypes in the MMHCdb, but the MGD does not provide information about the detailed characteristics of cancer models. For example, it is possible to search the MGD to find genotypes associated with increased or decreased incidence or susceptibility to specific cancers, but information on the tumor types that are typically observed in a cancer model and their frequency is information that is unique to the MMHCdb. The Mouse Phenome Database (MPD) (Bogue et al., 2023) and the International Mouse Phenotyping Consortium database (IMPC) (Groza et al., 2023) store baseline phenotype data collected from standardized phenotyping pipelines for different mouse strains and for genetically engineered lines of mice, but neither resource has substantial data for cancer phenotypes. Although the patient-derived models in MMHCdb are limited to those available from The Jackson Laboratory, MMHCdb collaborates with the European Bioinformatics Institute on the Patient-Derived Cancer Model Finder resource (PDCM) (Perova et al., 2022). The PDCM compiles information on patient-derived cancer models from repositories around the world and currently indexes over 4800 PDX models for more than 400 cancer types.

The MMHCdb currently includes data on over 60,000 mouse tumor models covering more than 1200 tumor classifications. These data have been acquired from more than 25,000 references and include over 7200 pathology records containing >6600 images with annotations. The images include 2596 JPGs and 3980 Zoomify TIFFs. The MMHCdb also contains 110 high-resolution whole-slide scans (Hamamatsu NDPI format) covering lung adenomas, lymphomas and leukemias. Whole-slide scans are presented online using the Open Microscopy Environment Remote Objects (OMERO) web-viewer. Pathology images in MMHCdb are made available to the research community for display with the permission of the submitting investigators or publishers. The cancer models in the MMHCdb are presented in over 110,000 tumor frequency records. Each of these records includes information on tissue of origin, tumor classification, frequency, genetic background and allelic composition. The MMHCdb also includes data on over 400 PDX models with more than 3000 histology and immunohistochemistry images.

## APPROACH

### Data acquisition

The two primary types of *in vivo* cancer models in the MMHCdb are mouse models (inbred strains and GEMMs) and human-in-mouse models (i.e. PDXs). A cancer model in MMHCdb is defined as a unique combination of organ of origin, tumor classification, organ affected, strain (background plus genotype) and tumor-inducing agent(s). Information and data in the MMHCdb are acquired from peer-reviewed scientific literature, direct submission by research laboratories, and through downloads from related resources including PathBase (Schofield et al., 2010) and the Gene Expression Omnibus (GEO) (Clough and Barrett, 2016). Publications with information relevant to MMHCdb are identified through the application of a machine-learning classifier that scans publications from more than 120 scientific journals and identifies papers that are likely to be relevant to the resource (Ringwald et al., 2022). All data in the MMHCdb are directly attributed to a reference, either a primary literature reference or a reference created for a submission or download, to identify the original source of the data.

### Nomenclature and ontology standards

Information and data acquired for MMHCdb are reviewed manually to ensure adherence to genetic nomenclature and annotation standards. Genes, alleles and mouse strains are named according to the rules established by the International Committee on Standardized Genetic Nomenclature for Mice. These entities are also linked to relevant records in the MGD, which provides users with MMHCdb information on additional phenotypes and disease models. Unofficial names and symbols used in publications are maintained as synonyms so that searches of MMHCdb using both official and unofficial nomenclature will return appropriate records.

Names of tumors in MMHCdb consist of two components: the organ of origin and a classification term (Bult et al., 2000). Both of the components rely on published community standards and terminologies, including Stedman's Medical Dictionary (Stedman et al., 1990), Pathology of the Mouse (Maronpot et al., 1999), Pathology of Tumours in Laboratory Animals, Volume II (Mohr and Turusov, 1994), International Classification of Rodent Tumors: The Mouse (Mohr, 2001), and Pathobiology of the Aging Mouse: Volumes 1 and 2 (Mohr et al., 1996a,b).

### Accessing MMHCdb

**Fig. 1.**
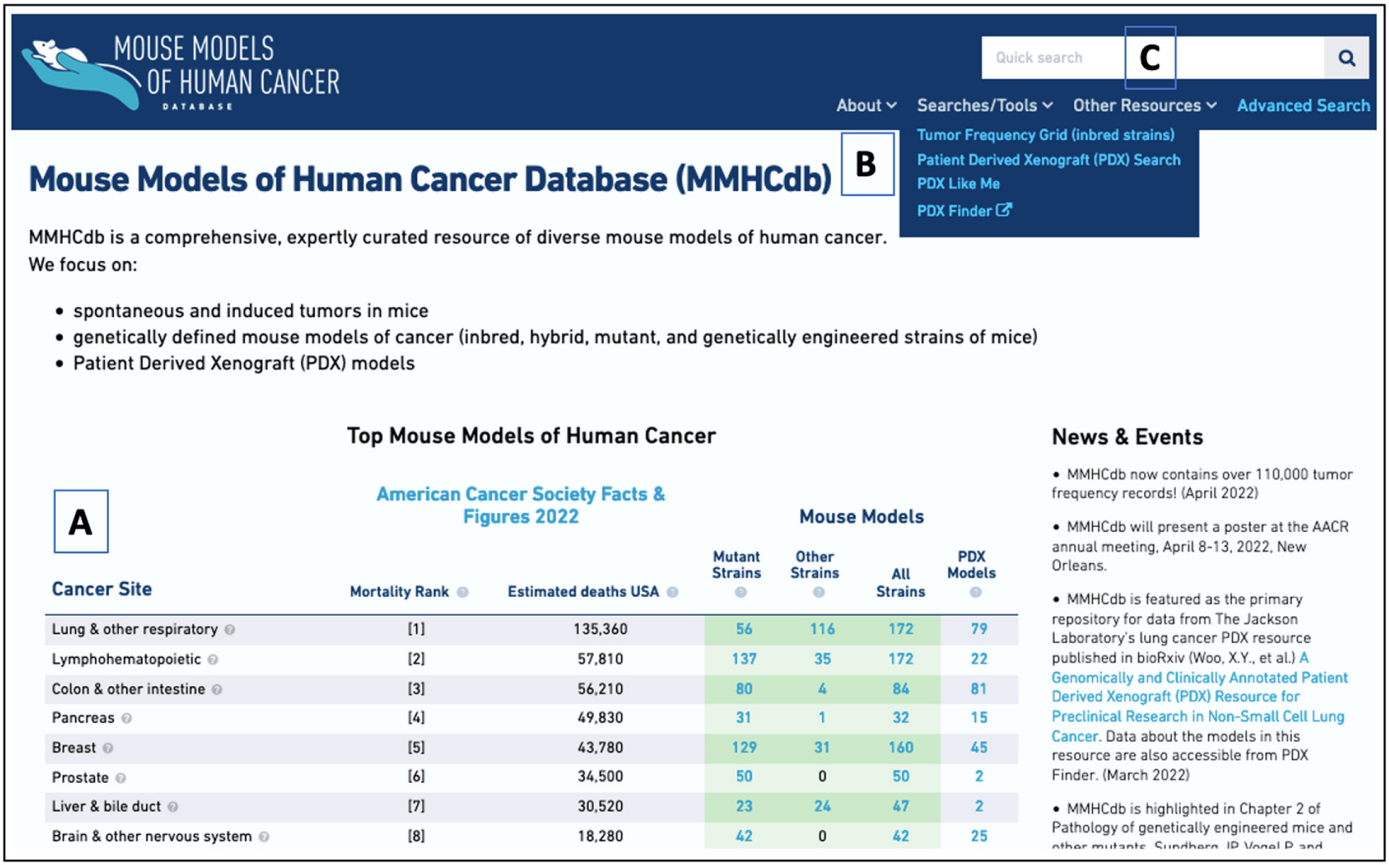
**The Mouse Models of Human Cancer database (MMHCdb).** The MMHCdb home page features a summary of different mouse strains associated with the top 20 human cancers, other database search options and a resource news feed. (A) Model summary table. (B) Menu access to search forms and data pages. (C) Quick search window.

The MMHCdb is freely available without registration. Summaries of mouse models for 20 cancer types with the highest mortality rates in humans are available via hypertext links from a human cancer overview table on the MMHCdb home page (Fig. 1). Also available from the home page are a Quick Search dialog box, a project-specific news feed, and links to other search tools and data resources (Fig. 1).

In addition to help documentation available from the ‘About Us’ menu, MMHCdb has a YouTube channel with short video tutorials about using the resource. Links to the instructional videos are available under the ‘Other Resources’ and ‘Help’ menus on the MMHCdb home page.

### Faceted searching

**Fig. 2.**
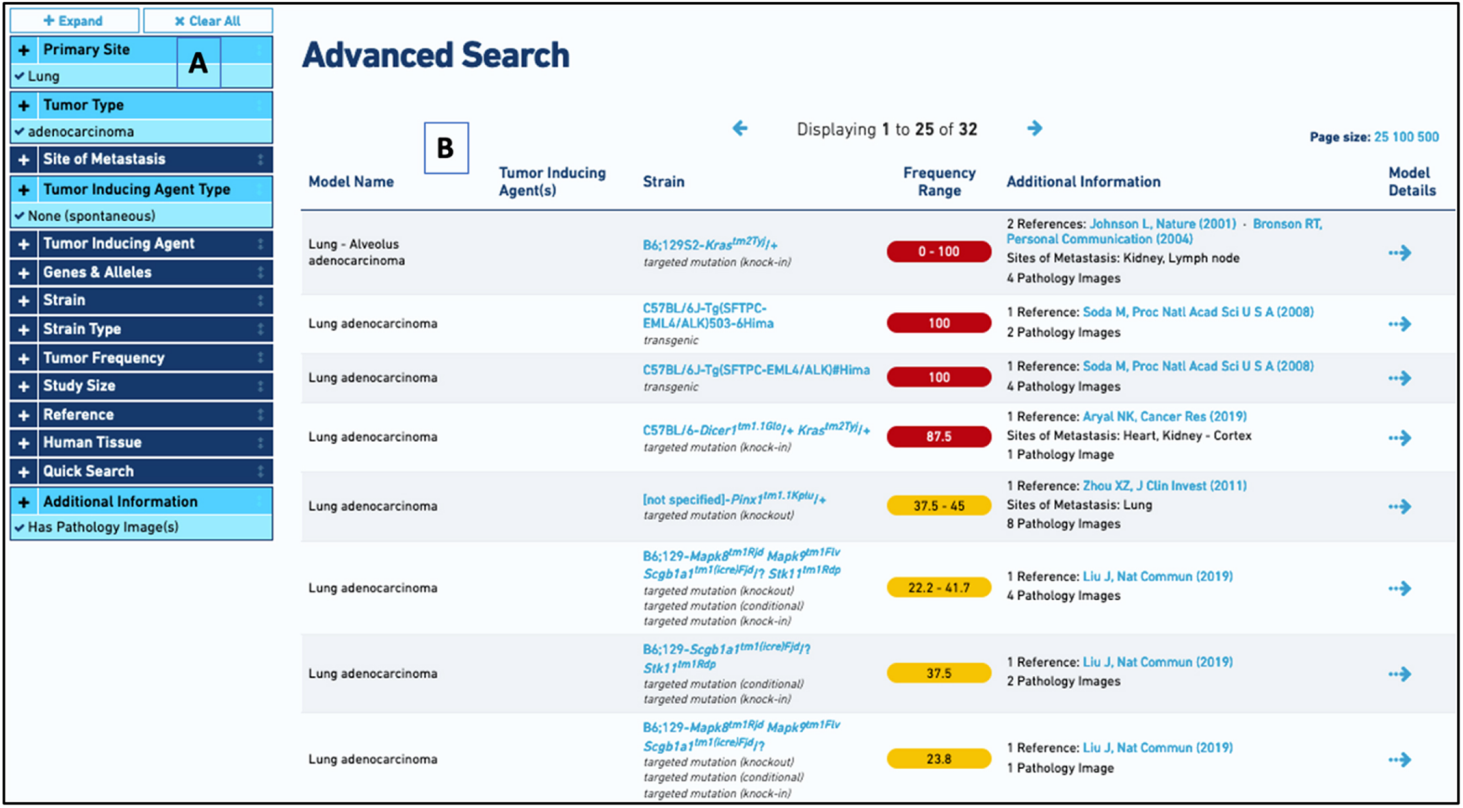
**Screenshot showing the faceted search function on the MMHCdb Advanced Search form for spontaneous lung adenocarcinoma models for which pathology images are available.** (A) The selected facets are shown in the left column, highlighted in light blue with a checkmark. (B) Search results summary with links to additional details about the strain, associated publications and model.

Faceted searching of MMHCdb is supported on the Advanced Search form. Search terms within each facet are presented as a picklist. Typing a term in the text box associated with each facet will narrow the list of terms using an autocomplete function. Search results update dynamically in response to changes in facet choices. The number of records a search term is associated with in the database is provided to give users a sense of the volume of data available. Search results include summary data for each matching model including model name, treatment status, strain name and type, tumor frequency range, and additional information or data available for the model. The results link to a detailed description of data available for a model and the publication(s) associated with the model. Strain names in MMHCdb are linked to reports that list all of the tumor models associated with the strain. An example of a faceted search for lung adenocarcinoma models for which there are pathology images available is shown in Fig. 2.

### Use cases

#### Use case 1: impact of genetic background on tumor types and frequencies

The genetic background of a mouse model can significantly affect the observed disease-related phenotypes, including the types and frequencies of tumors that are characteristic of a cancer model (Doetschman, 2009; Montagutelli, 2000). The same allele on different backgrounds can result in very different cancer characteristics and, therefore, impact the choice of model for a specific research application. For example, a human *HRAS* transgene, Tg(Wap-HRAS)69Lln, expressed on a mixed C57BL/6 and SJL background, subline 69-2, results in mammary gland carcinomas at a frequency of 45-50% by 1 year of age (Nielsen et al., 1991; Nielsen et al., 1995). However, mice carrying the same transgene expressed on an inbred FVB/N strain background, subline 69-2 crossed to FVB/N for two generations creating subline 69-2F, develop mammary gland tumors at a frequency of 100% by 3 months of age (Nielsen et al., 1995). On a C57BL/6J background, 100% of mice heterozygous for the *Apc^Min^* allele develop tumors throughout the intestine, particularly in the small intestine. When crossed onto an FVB/NJ background, only 7% of *Apc^Min^* heterozygous mice develop intestinal tumors (Svendsen et al., 2011). Mice heterozygous for the *Trp53^tm1Tyj^* allele develop mammary tumors on the BALB/c background, but not the C57BL/6J background (Reilly, 2016). In a survey of breast cancer models based on the mammary tumor virus promoter-driven polyoma middle T oncogene [Tg(MMTV-PyVT)634Mul], the parental transgenic FVB/N was crossed to 27 wild-type inbred strain backgrounds. The metastatic burden for the F1 progeny of these crosses varied by 40-fold depending on the background (Hunter et al., 2018; Lifsted et al., 1998).

**Fig. 3.**
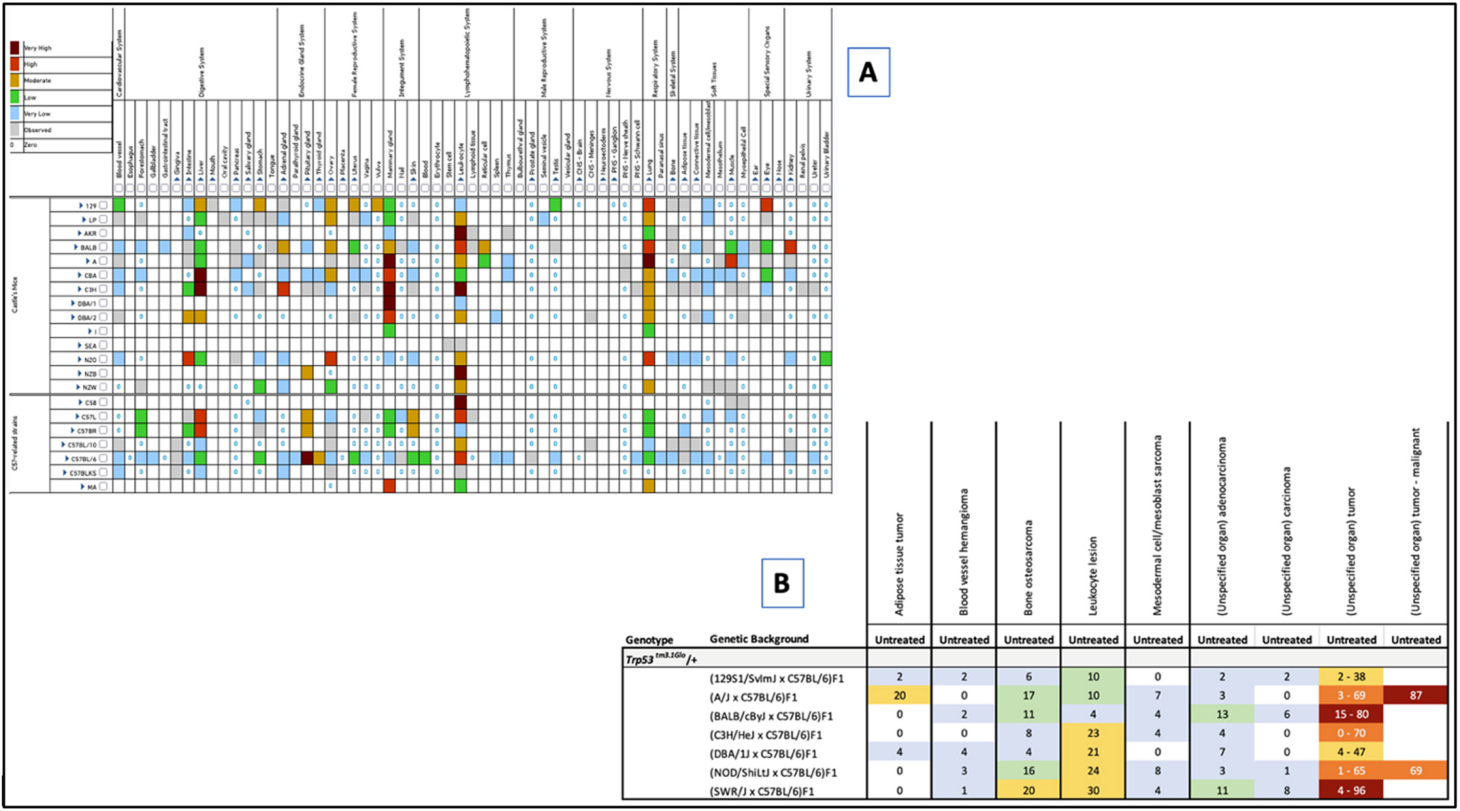
**Genetic background influences cancer phenotypes.** (A) A graphical summary of spontaneous tumor frequency in inbred strains is available from the Searches/Tools menu. (B) A summary of variation in types and frequency of tumor types among F1 hybrids heterozygous for the *Trp53^Tm3.1Glo^* allele. Color coding highlights the differences in cancer phenotypes for the same allele on different genetic backgrounds. Data from Chan et al. (2021).

As important as genetic background is in identifying appropriate disease models, finding this information through searches of the primary literature is time consuming and error prone. In the MMHCdb, users can quickly review the impact of genetic background on cancer phenotypes in two ways. First, a curated summary table of the frequency of spontaneous tumors for inbred strains from published and unpublished sources is available under the Searches/Tools menu on the home page (Fig. 3A). Second, data from curated publications that specifically mention the impact of genetic background are presented as a summary table with color coding of reported tumor frequency. Fig. 3B shows the results from a survey of tumor susceptibility in which tumor type and frequency were documented for F1 offspring of mice homozygous for *Trp53^tm3.1Glo^* on a C57BL/6 background that were crossed to seven different wild-type backgrounds (Chan et al., 2021; Levine, 2017). The survey results indicated that 21-30% of C3H, DBA, NOD and SWR F1 hybrid mice developed lymphomas/lymphoid hyperplasia, while only 4% of BALB/c F1 hybrids were observed to have this pathology. Twenty percent of A/J F1 hybrid mice developed lipomas, which were rarely observed in other backgrounds.

#### Use case 2: susceptibility to gastric cancer using Collaborative Cross mice

**Fig. 4.**
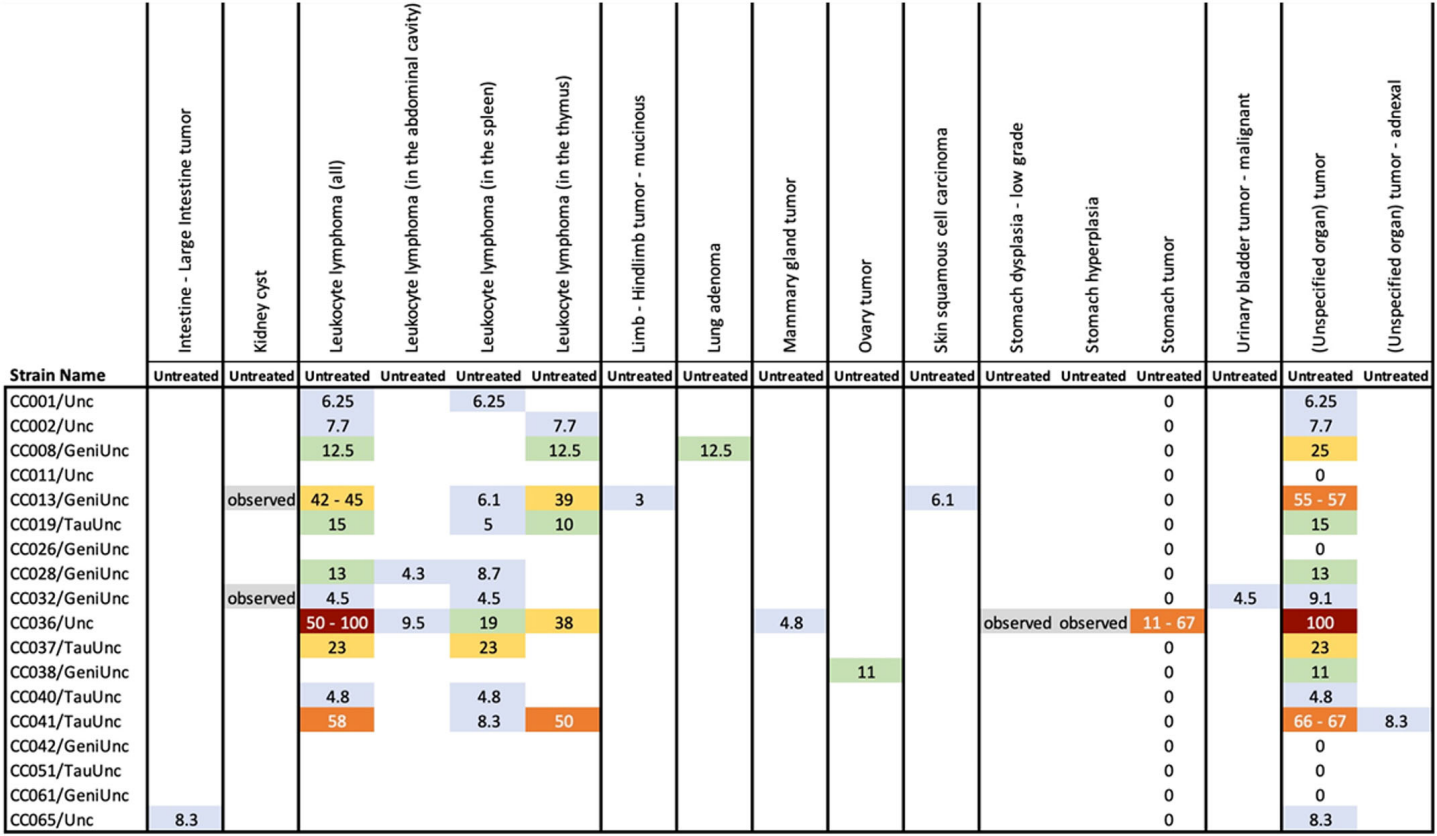
**MMHCdb-generated summary of cancer profiles for Collaborative Cross mice reported in Wang et al. (2019) (Stedman et al., 1990).** Wang et al. (2019) identified a novel Collaborative Cross line with increased susceptibility to gastric cancer (CC036/Unc).

The Collaborative Cross is a genetically diverse panel of recombinant inbred lines created by repeated crossing of eight inbred founder strains, which collectively comprise nearly 90% of the known genetic variation present in laboratory mice (Threadgill et al., 2011; Reilly, 2016). Collaborative Cross mice are a particularly useful experimental resource for identifying disease modifiers and genes associated with variation in cancer susceptibility. Wang et al. (2019) used 18 Collaborative Cross mice strains to examine the variation in tumor incidence and spectrum between strains, which led to the identification of a novel model for gastric cancer. In the MMHCdb, users can search for results on Collaborative Cross studies using the Strain Type=Collaborative Cross facet on the Advanced Search form. Fig. 4 shows the curated summary table for the Wang et al. (2019) study, revealing that gastric tumors were detected in one of the 18 Collaborative Cross strains examined (CC0036/Unc).

#### Use case 3: PDX models

PDXs have been used extensively for preclinical efficacy studies of single agent and combination cancer therapies (Kopetz et al., 2012; Lai et al., 2017). PDXs are generated through orthotopic or subcutaneous implantation of human tumor tissue into transplant-compliant immunodeficient mouse hosts (Ireson et al., 2019). The over 400 PDX models currently represented in the MMHCdb were generated by The Jackson Laboratory PDX Resource. These, and thousands of other PDX models from repositories around the world, are also included in the PDCM database that is maintained as a collaboration between MMHCdb and the European Bioinformatics Institute (EBI) (Perova et al., 2022). Deidentified model information such as age, sex and race of patient, host mouse, implantation method, tumor diagnosis and location, pathology annotations and images, and immunohistochemistry data are included. The MMHCdb also contains links to data repositories of genomic data such as the GEO, when available, including information on genomic sequence, copy-number variants, gene expression and tumor mutation burden. In addition, the PDX data also include preclinical study data, including treatment regimens and growth curves represented in multiple graphical methods.

**Fig. 5.**
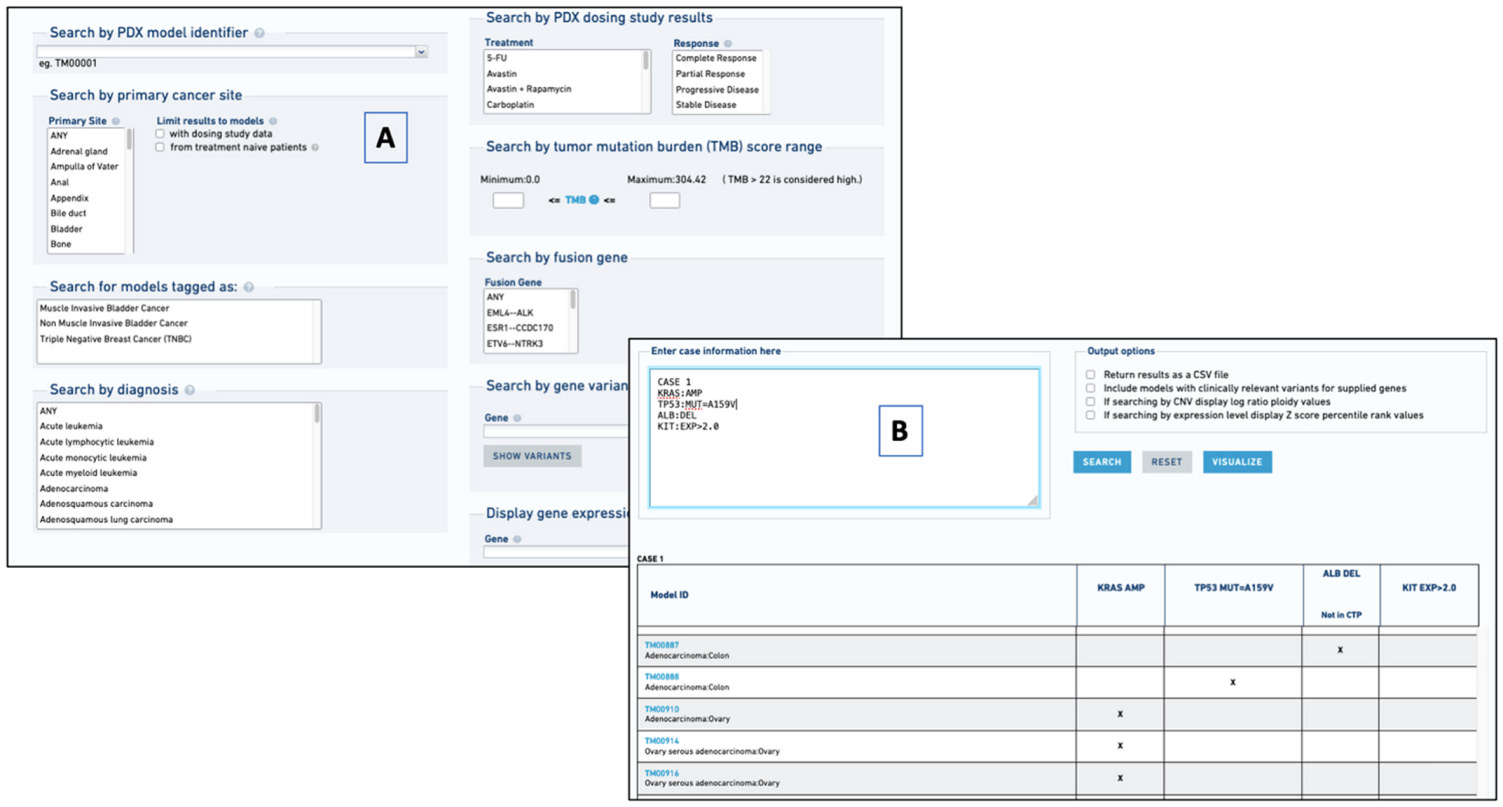
**Screenshots from the patient-derived xenograft (PDX) Portal.** (A) The PDX search form allows searching for PDX models using criteria such as tumor type, diagnosis, treatment response and genomic property. (B) The PDX Like Me query language finds models that match one or more molecular genomic criteria. PDX Like Me is modeled after the Onco Query Language in cBioPortal (Cerami et al., 2012).

Users can search for PDX models in the MMHCdb using web forms on the PDX Search Portal (Fig. 5A). Search criteria supported include model identifiers, cancer type, organ system, treatment results, tumor genome properties and allelic variants. Alternatively, users can search for models that match multiple molecular criteria using the ‘PDX Like Me’ query language (Fig. 5B). PDX Like Me is modeled after the cBioPortal's Onco Query Language (Cerami et al., 2012). Fig. 5B shows the results of a search for PDX models that have amplified *KRAS*, a *TP53 A159V* mutation, a deletion of the *ALB* gene, and high expression of the *KIT* gene.

### MMHCdb implementation details

The technical infrastructure that supports MMHCdb has four major software components described briefly below. The MMHCdb source code is available from GitHub repositories on Web interface, Database backend and Editorial interface.

#### Public web interface

The public web interface for MMHCdb runs on Apache HTTP server and Apache Tomcat. The web interface utilizes Java, Struts, JSP, JavaScript and HTML. Solr is used for the faceted advanced search. The EXT JS framework and Google Charts are used for interactive tables and graphics.

#### Curation interface

The primary curatorial interface for data entry is implemented as a Java Swing desktop application.

#### Database backend

The backend for MMHCdb is a highly normalized relational database running on Postgres.

#### Application programming interface

The MMHCdb application programming interface (API) is implemented as JSON-based web services. The APIs allow access to the MMHCdb data in a platform and language-independent manner that is sufficiently flexible to serve the diverse needs of bioinformaticians.

The MMHCdb was implemented in Java, Swing and Struts. These are mature technologies that are widely used, have a well-maintained codebase and are stable. New technologies are evaluated regularly as the functional features of the database evolve over time.

## DISCUSSION

The MMHCdb serves as an expertly curated knowledgebase for mouse models of human cancer. The two primary goals of the resource are to (1) facilitate aggregation of heterogeneous information generated by different laboratories for the same model through the enforcement of nomenclature and metadata annotation standards, and (2) highlight the impact of genetic background on the variation in the types of tumors and in the frequency of those types typical for a specific model.

*In vivo* mouse models have deeply informed our understanding of cancer biology, and the nature and use of these models is constantly evolving. Inbred mice and mice with spontaneous or chemically induced mutations have been used to study the basic genetic principles of gene function in cancer for many years (Reilly, 2016). Advancements in cellular and genome engineering technologies support the rapid generation of models carrying targeted mutations and conditional alleles with tissue- and temporal-specific expression of genes (Puccini et al., 2013; Guerin et al., 2020; Ran et al., 2013). PDX models have proven to be an exceptional platform for preclinical evaluation of the efficacy of novel cancer treatments (Kopetz et al., 2012). Mouse genetic diversity resources such as the Collaborative Cross are proving to be a powerful resource for investigating the genetic basis of cancer susceptibility, as well as susceptibility to adverse cancer treatment responses (Wang et al., 2019; Zeiss et al., 2019). Future plans for the MMHCdb include the continued adaptation of the resource to accommodate new types of mouse models of cancer as well as the implementation of tools and interfaces to support comparisons of mouse and human tumor genomics and treatment response data. These efforts will ensure that the MMHCdb continues to serve as a unique resource supporting research into the basic biology and genetics of human cancer and translational research.

## References

[DMM050001C1] Abate-Shen, C. and Pandolfi, P. P. (2013). Effective utilization and appropriate selection of genetically engineered mouse models for translational integration of mouse and human trials. *Cold Spring Harb. Protoc.* pdb.top078774. 10.1101/pdb.top078774 24173311 PMC4382078

[DMM050001C2] Blake, J. A., Baldarelli, R., Kadin, J. A., Richardson, J. E., Smith, C. L., Bult, C. J. and Mouse Genome Database Group (2021). Mouse Genome Database (MGD): Knowledgebase for mouse-human comparative biology. *Nucleic Acids Res.* 49, D981-D987. 10.1093/nar/gkaa1083 33231642 PMC7779030

[DMM050001C3] Bogue, M. A., Ball, R. L., Philip, V. M., Walton, D. O., Dunn, M. H., Kolishovski, G., Lamoureux, A., Gerring, M., Liang, H., Emerson, J. et al. (2023). Mouse Phenome Database: towards a more FAIR-compliant and TRUST-worthy data repository and tool suite for phenotypes and genotypes. *Nucleic Acids Res.* 51, D1067-D1074. 10.1093/nar/gkac1007 36330959 PMC9825561

[DMM050001C4] Bult, C. J., Krupke, D. M. and Eppig, J. T. (1999). Electronic access to mouse tumor data: the Mouse Tumor Biology Database (MTB) project. *Nucleic Acids Res.* 27, 99-105. 10.1093/nar/27.1.99 9847151 PMC148106

[DMM050001C5] Bult, C. J., Krupke, D. M., Begley, D. A., Richardson, J. E., Neuhauser, S. B., Sundberg, J. P. and Eppig, J. T. (2015). Mouse Tumor Biology (MTB): a database of mouse models for human cancer. *Nucleic Acids Res.* 43, D818-D824. 10.1093/nar/gku987 25332399 PMC4384039

[DMM050001C6] Bult, C. J., Krupke, D. M., Sundberg, J. P. and Eppig, J. T. (2000). Mouse tumor biology database (MTB): enhancements and current status. *Nucleic Acids Res.* 28, 112-114. 10.1093/nar/28.1.112 10592196 PMC102417

[DMM050001C7] Cerami, E., Gao, J., Dogrusoz, U., Gross, B. E., Sumer, S. O., Aksoy, B. A., Jacobsen, A., Byrne, C. J., Heuer, M. L., Larsson, E. et al. (2012). The cBio cancer genomics portal: an open platform for exploring multidimensional cancer genomics data. *Cancer Discov* 2, 401-404. 10.1158/2159-8290.CD-12-0095 22588877 PMC3956037

[DMM050001C8] Chan, C. S., Sun, Y., Ke, H., Zhao, Y., Belete, M., Zhang, C., Feng, Z., Levine, A. J. and Hu, W. (2021). Genetic and stochastic influences upon tumor formation and tumor types in Li-Fraumeni mouse models. *Life Sci Alliance* 4, e202000952. 10.26508/lsa.202000952 33376133 PMC7772779

[DMM050001C9] Chen, L., Liu, H. and Friedman, C. (2005). Gene name ambiguity of eukaryotic nomenclatures. *Bioinformatics* 21, 248-256. 10.1093/bioinformatics/bth496 15333458

[DMM050001C10] Clough, E. and Barrett, T. (2016). The gene expression omnibus database. *Methods Mol. Biol.* 1418, 93-110. 10.1007/978-1-4939-3578-9_5 27008011 PMC4944384

[DMM050001C11] Doetschman, T. (2009). Influence of genetic background on genetically engineered mouse phenotypes. *Methods Mol. Biol.* 530, 423-433. 10.1007/978-1-59745-471-1_23 19266333 PMC2805848

[DMM050001C12] Groza, T., Gomez, F. L., Mashhadi, H. H., Muñoz-Fuentes, V., Gunes, O., Wilson, R., Cacheiro, P., Frost, A., Keskivali-Bond, P., Vardal, B. et al. (2023). The International Mouse Phenotyping Consortium: comprehensive knockout phenotyping underpinning the study of human disease. *Nucleic Acids Res.* 51, D1038-D1045. 10.1093/nar/gkac972 36305825 PMC9825559

[DMM050001C13] Guerin, M. V., Finisguerra, V., Van Den Eynde, B. J., Bercovici, N. and Trautmann, A. (2020). Preclinical murine tumor models: a structural and functional perspective. *Elife* 9, e50740. 10.7554/eLife.50740 31990272 PMC6986875

[DMM050001C14] Hunter, K. W., Amin, R., Deasy, S., Ha, N. H. and Wakefield, L. (2018). Genetic insights into the morass of metastatic heterogeneity. *Nat. Rev. Cancer* 18, 211-223. 10.1038/nrc.2017.126 29422598 PMC6290469

[DMM050001C15] Ireson, C. R., Alavijeh, M. S., Palmer, A. M., Fowler, E. R. and Jones, H. J. (2019). The role of mouse tumour models in the discovery and development of anticancer drugs. *Br. J. Cancer* 121, 101-108. 10.1038/s41416-019-0495-5 31231121 PMC6738037

[DMM050001C16] Justice, M. J., Siracusa, L. D. and Stewart, A. F. (2011). Technical approaches for mouse models of human disease. *Dis. Model. Mech.* 4, 305-310. 10.1242/dmm.000901 21558063 PMC3097452

[DMM050001C17] Kopetz, S., Lemos, R. and Powis, G. (2012). The promise of patient-derived xenografts: the best laid plans of mice and men. *Clin. Cancer Res.* 18, 5160-5162. 10.1158/1078-0432.CCR-12-2408 22912394 PMC4217576

[DMM050001C18] Lai, Y., Wei, X., Lin, S., Qin, L., Cheng, L. and Li, P. (2017). Current status and perspectives of patient-derived xenograft models in cancer research. *J. Hematol. Oncol.* 10, 106. 10.1186/s13045-017-0470-7 28499452 PMC5427553

[DMM050001C19] Levine, A. J. (2017). The evolution of tumor formation in humans and mice with inherited mutations in the p53 gene. *Curr. Top. Microbiol. Immunol.* 407, 205-221. 10.1007/82_2017_5 28349284 PMC6383363

[DMM050001C20] Lifsted, T., Le Voyer, T., Williams, M., Muller, W., Klein-Szanto, A., Buetow, K. H. and Hunter, K. W. (1998). Identification of inbred mouse strains harboring genetic modifiers of mammary tumor age of onset and metastatic progression. *Int. J. Cancer* 77, 640-644. 10.1002/(SICI)1097-0215(19980812)77:4<640::AID-IJC26>3.0.CO;2-8 9679770

[DMM050001C21] Little, C. C. and Tyzzer, E. E. (1916). Further experimental studies on the inheritance of susceptibility to a Transplantable tumor, Carcinoma (J. W. A.) of the Japanese waltzing Mouse. *J Med Res* 33, 393-453. 19972275 PMC2083849

[DMM050001C22] Liu, E. T., Bolcun-Filas, E., Grass, D. S., Lutz, C., Murray, S., Shultz, L. and Rosenthal, N. (2017). Of mice and CRISPR: The post-CRISPR future of the mouse as a model system for the human condition. *EMBO Rep.* 18, 187-193. 10.15252/embr.201643717 28119373 PMC5286389

[DMM050001C23] Maronpot, R., Boorman, G. and Gaul, B. (1999). *Pathology of the Mouse: Reference and Atlas*, p. 699. Cache River Pr.

[DMM050001C24] Mcgonigle, P. and Ruggeri, B. (2014). Animal models of human disease: challenges in enabling translation. *Biochem. Pharmacol.* 87, 162-171. 10.1016/j.bcp.2013.08.006 23954708

[DMM050001C25] Mohr, U. (2001). *International Classification of Rodent Tumors: The Mouse*, p. 474. Springer Nature.

[DMM050001C26] Mohr, U., Dungworth, D., Ward, J., Capen, C., Carlton, W. and Sundberg, J. (1996a). *Pathobiology of the Aging Mouse*, Vol. 1. International Life Sciences Institute (ILSI) Press.

[DMM050001C27] Mohr, U., Dungworth, D., Ward, J., Capen, C., Carlton, W. and Sundberg, J. (1996b). *Pathobiology of the Aging Mouse*, Vol. 2, p. 505. International Life Sciences Institute (ILSI) Press.

[DMM050001C28] Mohr, U. and Turusov, V. (1994). *Pathology of Tumours in Laboratory Animals Vol. II: Tumours of the Mouse*, p. 800. Oxford University Press.

[DMM050001C29] Montagutelli, X. (2000). Effect of the genetic background on the phenotype of mouse mutations. *J. Am. Soc. Nephrol.* 11 Suppl. 16, S101-S105. 10.1681/ASN.V11suppl_2s101 11065339

[DMM050001C30] Naf, D., Krupke, D. M., Sundberg, J. P., Eppig, J. T. and Bult, C. J. (2002). The Mouse Tumor Biology Database: a public resource for cancer genetics and pathology of the mouse. *Cancer Res.* 62, 1235-1240. 11888882

[DMM050001C31] Nielsen, L. L., Discafani, C. M., Gurnani, M. and Tyler, R. D. (1991). Histopathology of salivary and mammary gland tumors in transgenic mice expressing a human Ha-ras oncogene. *Cancer Res.* 51, 3762-3767. 2065330

[DMM050001C32] Nielsen, L. L., Gurnani, M., Catino, J. J. and Tyler, R. D. (1995). In wap-ras transgenic mice, tumor phenotype but not cyclophosphamide-sensitivity is affected by genetic background. *Anticancer Res.* 15, 385-392. 7763010

[DMM050001C33] Perova, Z., Martinez, M., Mandloi, T., Lopez Gomez, F., Halmagyi, C., Follette, A., Mason, J., Newhauser, S., Begley, D. A., Krupke, D. M. et al. (2022). PDCM Finder: an open global research platform for patient-derived cancer models. *Nucleic Acids Res.* 51, D1360-D1366. 10.1093/nar/gkac1021 PMC9825610 36399494

[DMM050001C34] Puccini, J., Dorstyn, L. and Kumar, S. (2013). Genetic background and tumour susceptibility in mouse models. *Cell Death Differ.* 20, 964. 10.1038/cdd.2013.35 23618812 PMC3679461

[DMM050001C35] Ran, F. A., Hsu, P. D., Wright, J., Agarwala, V., Scott, D. A. and Zhang, F. (2013). Genome engineering using the CRISPR-Cas9 system. *Nat. Protoc.* 8, 2281-2308. 10.1038/nprot.2013.143 24157548 PMC3969860

[DMM050001C36] Reilly, K. M. (2016). The effects of genetic background of mouse models of cancer: friend or foe? *Cold Spring Harb Protoc* 2016, pdb top076273. 10.1101/pdb.top076273 26933251 PMC6703156

[DMM050001C37] Ringwald, M., Richardson, J. E., Baldarelli, R. M., Blake, J. A., Kadin, J. A., Smith, C. and Bult, C. J. (2022). Mouse Genome Informatics (MGI): latest news from MGD and GXD. *Mamm. Genome* 33, 4-18. 10.1007/s00335-021-09921-0 34698891 PMC8913530

[DMM050001C38] Schofield, P. N., Gruenberger, M. and Sundberg, J. P. (2010). Pathbase and the MPATH ontology. Community resources for mouse histopathology. *Vet. Pathol.* 47, 1016-1020. 10.1177/0300985810374845 20587689 PMC3038412

[DMM050001C39] Sharpless, N. E. and Depinho, R. A. (2006). The mighty mouse: genetically engineered mouse models in cancer drug development. *Nat. Rev. Drug Discov.* 5, 741-754. 10.1038/nrd2110 16915232

[DMM050001C40] Stedman, T., Williams, R. and Stedman, J. (1990). *Stedman's Medical Dictionary*, p. 95. Williams & Wilkins.

[DMM050001C41] Sundberg, J. P., Roopenian, D. C., Liu, E. T. and Schofield, P. N. (2013). The Cinderella effect: searching for the best fit between mouse models andhuman diseases. *J. Invest. Dermatol.* 133, 2509-2513. 10.1038/jid.2013.238 23812235

[DMM050001C42] Svendsen, C., Alexander, J., Knutsen, H. K. and Husoy, T. (2011). The min mouse on FVB background: susceptibility to spontaneous and carcinogen-induced intestinal tumourigenesis. *Anticancer Res.* 31, 785-788. 21498697

[DMM050001C43] Threadgill, D. W., Miller, D. R., Churchill, G. A. and De Villena, F. P. (2011). The collaborative cross: a recombinant inbred mouse population for the systems genetic era. *ILAR J.* 52, 24-31. 10.1093/ilar.52.1.24 21411855

[DMM050001C44] Tyzzer, E. E. (1909). A series of spontaneous tumors in mice with observations on the influence of heredity on the frequency of their occurrence. *J Med Res* 21, 479-518.13. 19971929 PMC2099039

[DMM050001C45] Wang, P., Wang, Y., Langley, S. A., Zhou, Y. X., Jen, K. Y., Sun, Q., Brislawn, C., Rojas, C. M., Wahl, K. L., Wang, T. et al. (2019). Diverse tumour susceptibility in Collaborative Cross mice: identification of a new mouse model for human gastric tumourigenesis. *Gut* 68, 1942-1952. 10.1136/gutjnl-2018-316691 30842212 PMC6839736

[DMM050001C46] Zeiss, C. J., Gatti, D. M., Toro-Salazar, O., Davis, C., Lutz, C. M., Spinale, F., Stearns, T., Furtado, M. B. and Churchill, G. A. (2019). Doxorubicin-induced cardiotoxicity in collaborative cross (CC) mice recapitulates individual cardiotoxicity in humans. *G3 (Bethesda)* 9, 2637-2646. 10.1534/g3.119.400232 31263061 PMC6686936

